# Changes of Plasma Tris(hydroxymethyl)aminomethane and 5-Guanidino-3-methyl-2-oxopentanoic Acid as Biomarkers of Heart Remodeling after Left Ventricular Assist Device Support

**DOI:** 10.3390/metabo12111068

**Published:** 2022-11-04

**Authors:** Mengda Xu, Hao Cui, Xiao Chen, Xiumeng Hua, Jiangping Song, Shengshou Hu

**Affiliations:** 1 Union Hospital, Tongji Medical College, Huazhong University of Science and Technology, Wuhan 430030, China; 2 State Key Laboratory of Cardiovascular Disease, Fuwai Hospital, Chinese Academy of Medical Sciences, Shenzhen 518057, China; 3 State Key Laboratory of Cardiovascular Disease, Fuwai Hospital, National Center for Cardiovascular Diseases, Chinese Academy of Medical Sciences and Peking Union Medical College, 167A Beilishi Road, Xi Cheng District, Beijing 100037, China

**Keywords:** untargeted metabolomic analysis, LVAD, biomarker, remodeling of cardiac function

## Abstract

Cardiac function is closely related to heart metabolism. Heart failure patients undergoing LVAD support have shown varying degrees of remodeling of both cardiac function and morphology. However, the metabolic changes in patients with different outcomes are unclear. This study aimed to identify metabolic differences and evaluate metabolomics-based biomarkers in patients with non-improved/improved cardiac function after LVAD support. Sixteen patients were enrolled in this study. Plasma samples were analyzed by using untargeted metabolomic approaches. Multivariate statistical analysis and a Mann–Whitney U-test was performed to clarify the separation in metabolites and to identify changes in plasma metabolites between the two groups, respectively. The efficacy of candidate biomarkers was tested by the area under the curve receiver operating characteristic curve. Using the Metabolomics Standards Initiative level 2, a total of 1542 and 619 metabolites were detected in the positive and negative ion modes, respectively. Enrichment analysis showed that metabolites in improved cardiac function patients were mainly involved in carbohydrate metabolism and amino acid metabolism. Metabolites from non-improved cardiac function patients were mainly involved in hormone metabolism. Furthermore, we found tris(hydroxymethyl)aminomethane and 5-guanidino-3-methyl-2-oxopentanoic acid could serve as biomarkers to predict whether a patient’s cardiac function would improve after LVAD support.

## 1. Introduction

Heart transplantation (HTx) is the most effective treatment for end-stage heart failure (HF). However, donor shortages, side effects of immunosuppressive drugs and graft failure currently limit the therapeutic benefits of HTx. The left ventricular assist device (LVAD) support alleviates these problems [1]. One study found that 21% of patients with non-ischemic cardiomyopathy and 5% of patients with ischemic cardiomyopathy showed varying degrees of remodeling in cardiac function (left ventricular ejection fraction, LVEF) and morphology (left ventricular end-diastolic diameter, LVEDD) during LVAD support [2]. These results suggested that patients undergoing LVAD support may have either improved cardiac function (ICF) or non-improved cardiac function (nICF), but the metabolic characteristics of patients with different outcomes were unclear.

Recently several studies have clarified that LVAD support could induce significant changes in the cardiac transcript, protein, and metabolism. On the transcriptional level, Nerbonne et al. performed RNA sequencing in HF patients undergoing LVAD support and found that lncRNA gradually returned to normal levels during the improvement of cardiac function. It was further hypothesized that lncRNAs might serve as important molecules to differentiate patients with different clinical outcomes [3]. By using microarray analysis, Thum et al. found that the concentration of plasma miR-483-3p was negatively correlated with the plasma level of NT-proBNP. Thus, miR-483-3p might be served as a biomarker of the efficacy of LVAD support. In addition, miR-1202 could be used as a predictive molecule for the degree of response to LVAD support [4]. On the protein level, studies have reported that reduced levels of immune system-related proteins, increased levels of membrane biology-related proteins as well as improved T-tubule structure were detected following LVAD support [5]. On the metabolism level, Drakos et al. found that levels of glucose transporter proteins were increased after LVAD support by measuring the levels of key enzymes and metabolic substrates associated with glucose metabolism in myocardial tissue [6]. The above results helped us to further understand the changes in transcriptome, proteome, and glucose metabolism after LVAD support. However, there are limitations in the above studies, such as the complex biological functions of non-coding RNAs and the unstable state of single-stranded RNAs, which can be easily degraded, resulting in less accurate detection results. In addition, most studies use myocardial tissue as the test sample, which is difficult to carry out in the clinical management of patients with LVAD support. In addition to the above issues, NT-proBNP is currently used as a biomarker to assess cardiac function in patients, but NT-proBNP is a substance that is compensated for elevated tension felt by the ventricles and does not predict changes in cardiac structure or function in advance. Therefore, there is a certain lag in using NT-proBNP as a biomarker to assess changes in cardiac function after LVAD support. As the structural and functional status of the heart was closely correlated to its metabolic profile, HF patients underwent significant changes in energy metabolism [7] in the context of the myocardial metabolic disorder prior to the onset of altered cardiac function [8]. Therefore, characterizing the metabolomic changes in patients after LVAD support could help in the early post-operative risk assessment and management of patients.

High-throughput metabolomics is a systematic approach for identifying small-molecule (<1500 Da) metabolite profiles that have important potential for predicting disease states [9]. Metabolomics is a way of quantifying all the metabolites in an organism, following the ideas of genomics and proteomics and finding the relative relationship between metabolites and physiological and pathological changes [10]. Mass Spectrometry (MS)-based metabolomics is highly selective and sensitive, capable of detecting multiple metabolites simultaneously, and has a wide range of applications. Several studies have been conducted on cardiovascular diseases through MS-based metabolomics and identified a variety of metabolites that can be used as predictors of disease progression. According to plasma metabolomics, phenylacetylglutamine was first identified to be associated with major cardiovascular adverse events (MACEs) in a discovery cohort of 1162 patients. It was then validated in a large cohort to cause an increase in MACEs. This led to its use as a biomarker for predicting MACEs [11]. In addition, metabolomic testing of plasma has identified elevated levels of gluconic acid, fumaric acid, and pseudouridine that may predict acute kidney injury following cardiac surgery [12]. These results demonstrate high throughput metabolomics is a powerful approach for developing novel biomarkers.

Based on the significant changes in energy metabolism in HF patients and alteration of metabolic substrate utilization after LVAD support, we propose to use an untargeted metabolomics approach to analyze pre- and post-operative plasma samples from LVAD support patients to identify changes in the metabolome and to screen for biomarkers that can predict improvements in cardiac function. These studies will guide the precise treatment of patients and fill the current gap in the field of LVAD research.

## 2. Materials and Methods

### 2.1. Study Design

Plasma samples were prospectively collected prior to the LVAD implantation and post-implantation, following at least 24h of LVAD support. Clinical data (including assessment of clinical symptoms, imaging, and hematology) were recorded at baseline and during the entire follow-up phase. Patients’ cardiac function underwent preoperatively and 3–6 month post-operative echocardiographic assessment to evaluate signs of improvement [6]. Patients with ICF should meet at least one of the following criteria: (1) having a relative increase in LVEF ≥ 50% compared to baseline; (2) a final LVEF ≥ 40%; (3) a final LVEDD < 6 cm. Patients who did not meet any of these criteria were classified as nICF.

### 2.2. Metabolite Extraction from Plasma Samples

Metabolite extraction was performed using a previously described protocol [13]. Briefly, 50 μL aliquots of plasma were added into 450 μL acetonitrile:methanol (*v/v*, 1:1), followed by vortexing for 5 min. The mixture was then centrifuged at 4 °C, 18,400× *g* for 30 min to exclude proteins. After the centrifugation, the supernatants were further processed for LC-MS/MS analysis. A mixture comprising equal volume from each sample was used as a quality control (QC) sample.

### 2.3. Untargeted LC-MS/MS-Based Metabolomic Analysis

Metabolite profiling was developed using a Vanquish ultra-performance liquid chromatography system coupled to a Q-Exactive HF mass spectrometer (Thermo Fisher Scientific, Waltham, MA, USA). Metabolite separation was performed at 40 °C in a Hypersil GOLD C18 column (100 × 2.1 mm, 1.9 μm, Thermo Fisher Scientific). A 15 min gradient at a flow rate of 0.25 mL/min was used to separate metabolites. Mobile phase A was H_2_O with 0.1% FA, and mobile phase B was ACN with 0.1% FA. The gradient was set as follows: 0–1.5 min, 5% B; 1.5–6.0 min, 5–95% B; 6.0–11.0 min, 95% B; 11.0–11.5 min, 95–5% B; 11.5–15.0 min, 5% B.

The MS was operated in electrospray ionization positive ion mode and negative ion mode. Analysis was performed in the full scan [mass-to-charge ratio (*m/z*) = 67~1000] and data-dependent scan (dd-MS^2^) modes (the parent ion ranked in the top five). The instrument settings for the full scan mode were: 120,000 resolution, 2 × 10^6^ automatic gain control (AGC), and 200 ms maximum ion injection time (IT). The settings for the MS/MS mode were: 30,000 resolution, 1 × 10^5^ AGC, 100 ms maximum IT, 15 s dynamic exclusion and collision energy of 40. Source ionization parameters were spray voltage set at 3.5 kV for positive ion mode and 4.0 kV for negative ion mode, capillary temperature set at 320 °C, sheath gas set at 25, and aux gas set at 5.

### 2.4. Data Processing

The chemical analysis working group of the Metabolomics Standards Initiative (MSI) defined four different levels of metabolite identification, which include identified metabolites (level 1), putatively annotated compounds (level 2), putatively characterized compound classes (level 3), and unknown compounds (level 4). In this study, we applied to level 2 annotation [14,15]. The raw data was processed using Compound Discoverer version 3.1 software (Thermo Fisher Scientific) with the manufacturer’s recommended parameters to conduct peak area extraction and metabolite identification. Molecular mass was initially used to search against the commercial database from Thermo Fisher Scientific (McCloud). Mass and adduct types were used to search the HMDB, KEGG and Biocyc. The m/z features with < 20% for missing values and spectral relative standard deviations < 30% for QC were included in subsequent statistical analyses.

### 2.5. Statistical Analysis

Differences in metabolite levels between ICF and nICF patients were assessed with Mann–Whitney U-tests. *p*-Values < 0.05 were considered to be statistically significant. Principle Component Analysis (PCA) and Orthogonal Partial least squares Discriminant Analysis (OPLS-DA) were performed using MetaboAnalyst version 5.0 (https://www.metaboanalyst.ca). Receiver operating characteristic (ROC) curve analyses were used for developing biomarkers, and their predictive abilities were tested using the area under the curve (AUC). Correlation analysis was performed using IBM SPSS version 20.0 (International Business Machines Corporation, Armonk, NY, USA).

## 3. Results

### 3.1. Demographic Characteristics of the Patients 

Patients’ general demographic and clinical data are presented in Table 1. According to the grouping criteria, 6 patients were classified as ICF, and the other 10 patients were classified as nICF. The mean age at implantation of ICF and nICF patients was 39.2 years (IQR:30.5–46.25 years) and 40.5 years (IQR:30.75–47.75 years). There were 6 male patients (100%) in the ICF group and 9 male patients (90%) in the nICF group. The causes of HF in the ICF group were ischemic cardiomyopathy in 2 patients (33%) and dilated cardiomyopathy in 4 patients (67%). In the meantime, the causes of HF in the nICF group were ischemic cardiomyopathy in 1 patient (10%), dilated cardiomyopathy in 6 patients (60%), valvular heart disease in 2 patients (20%) and alcoholic cardiomyopathy in 1 patient (10%). There was no significant difference in the above parameters between the two groups. Furthermore, body mass index, New York Heart Association heart function, serum creatinine, hemoglobin and diabetes were not significantly different between the two groups.

### 3.2. Distinguishing ICF Patients from nICF Patients Using Plasma Metabolomics

**Figure 1:**
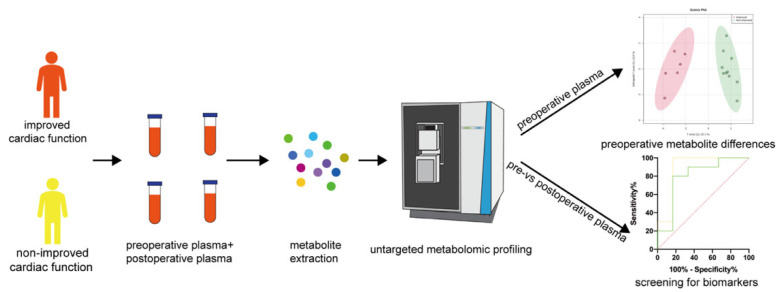
Schematic workflow of the present study.

Untargeted metabolomics was performed in both pre- and post-operative plasma samples to clarify the changes in metabolites between the two groups and identify potential biomarkers to predict the changes in cardiac function (Figure 1). 

**Figure 2:**
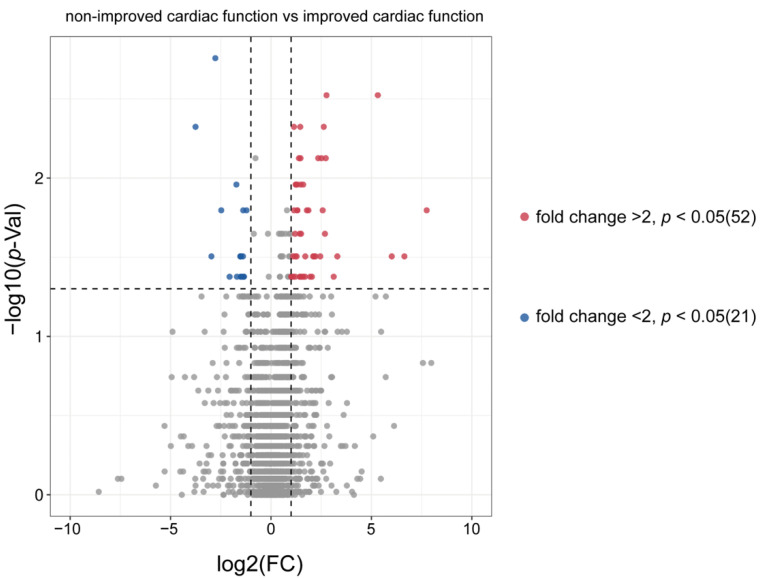
Volcano plot of metabolic profiles in preoperative plasma from non-improved cardiac function patients and improved cardiac function patients: 52 metabolites were significantly (*p* < 0.05) upregulated (fold change > 2) in non-improved cardiac function patients and 21 metabolites were significantly (*p* < 0.05) downregulated (fold change < 2) in non-improved cardiac function patients.

A total of 1542 and 619 metabolites were detected in the positive and negative ion modes, respectively. There were 96 metabolites significantly different between nICF patients and ICF patients, 52 metabolites showed a fold change (FC) > 2, and 21 metabolites showed an FC < 2 between nICF patients and ICF patients (Figure 2 and Table S1). 

**Figure 3:**
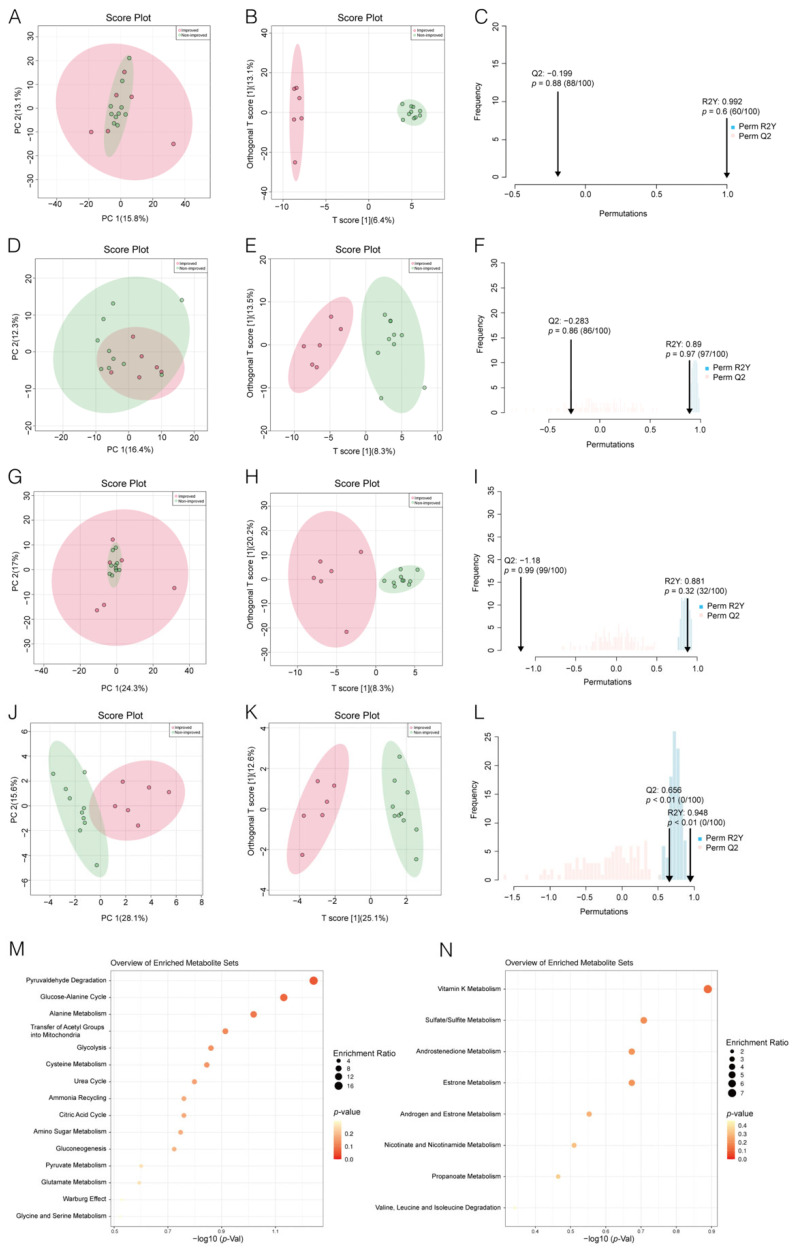
Separation and classification of metabolic profiles in preoperative plasma from non-improved cardiac function (nICF) patients and improved cardiac function (ICF) patients: (**A**). Principle Component Analysis (PCA) score plots based on 714 endogenous metabolites. (**B**). Orthogonal Partial least squares Discriminant Analysis (OPLS-DA) score plots based on 714 endogenous metabolites. (**C**). Permutation test of OPLS-DA score plots based on 714 endogenous metabolites. (**D**). PCA score plots based on metabolites that were upregulated in nICF patients. (**E**). OPLS-DA score plots based on metabolites that were upregulated in nICF patients. (**F**). Permutation test of OPLS-DA score plots based on metabolites that were upregulated in nICF patients. (**G**). PCA score plots based on metabolites that were downregulated in nICF patients. (**H**). OPLS-DA score plots based on metabolites that were downregulated in nICF patients. (**I**). Permutation test of OPLS-DA score plots based on metabolites that were downregulated in nICF patients. (**J**). PCA score plots based on metabolites that were significantly different between nICF patients and ICF patients. (K). OPLS-DA score plots based on metabolites were significantly different between nICF patients and ICF patients. (**L**). Permutation test of OPLS-DA score plots based on metabolites that were significantly different between nICF patients and ICF patients. (**M**). Enrichment analysis based on metabolites that were significantly upregulated in ICF patients. (**N**). Enrichment analysis based on metabolites that were significantly upregulated in nICF patients.

Subsequently, based on the largest database of human metabolites-the human metabolome database (HMDB, https://hmdb.ca), we finally identified 714 endogenous metabolites (Table S2). PCA was used to identify the general characteristic of ICF and nICF patients. The 714 endogenous metabolites were entered into MetaboAnalyst version 5.0 to establish a PCA model. Score plots showed that there was no obvious separation between the two groups of patients (Figure 3A). 

Furthermore, we performed an OPLS-DA analysis to identify the potential difference between the ICF patients and nICF patients. Optimum separation of the metabolic profiles for ICF patients and nICF patients was achieved from the OPLS-DA score plots (Figure 3B). However, when the permutation test was performed, Q2 was less than 0 (−0.199, *p* = 0.88), suggesting an overfitting of the OPLS-DA model (Figure 3C). To maximize the identification of the metabolite profile in ICF and nICF patients, we then performed the PCA and OPLS-DA analyses for the metabolites upregulated in nICF patients (Figure 3D–F) and metabolites downregulated in nICF patients (Figure 3G–I), both showing that there was no obvious separation of the score plots for PCA or overfitting of the OPLS-DA model. The above results indicated that it was difficult to clarify the difference between ICF patients and nICF patients based on metabolites of preoperative plasma. Finally, we used a total of 24 metabolites that were significantly different between the two groups to perform PCA and OPLS-DA (Table S3). Optimum separation of the metabolic profiles for nICF patients and ICF patients was achieved from both the PCA score plots and OPLS-DA score plots (R2Y, 0.948; Q2, 0.656) (Figure 3J–L). Based on these results, we further performed a functional enrichment analysis of metabolites that were significantly different between the two groups, and the results showed that the metabolites that were significantly elevated in the ICF patients were mainly enriched in carbohydrate metabolism (glycolysis, gluconeogenesis, pyruvate metabolism), amino acid metabolism (glucose-alanine cycle, cysteine metabolism, glycine and serine metabolism and alanine metabolism) and ATP metabolism (citric acid cycle) (Figure 3M), whereas the metabolites that were significantly elevated in the nICF patients were mainly enriched in hormone metabolism (androstenedione metabolism and estrone metabolism) (Figure 3N).

### 3.3. Identification of Potential Biomarkers

**Figure 4:**
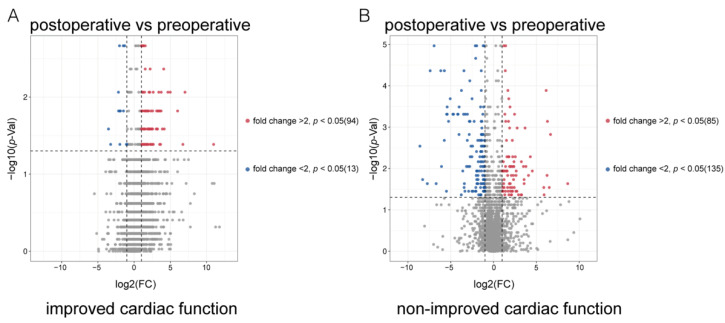
Volcano plot of metabolic profiles in preoperative plasma and post-operative plasma from non-improved cardiac function (nICF) patients and improved cardiac function (ICF) patients: (**A**). Ninety-four metabolites were significantly (*p* < 0.05) upregulated (fold change > 2) in ICF patients after LVAD support, and 13 metabolites were significantly (*p* < 0.05) downregulated (fold change < 2) in ICF patients after LVAD support. (**B**). Eighty-five metabolites were significantly (*p* < 0.05) upregulated (fold change > 2) in nICF patients after LVAD support, and 135 metabolites were significantly (*p* < 0.05) downregulated (fold change < 2) in nICF patients after LVAD support.

To further develop predictive biomarkers from the metabolites, we calculated the extent to which the patients’ preoperative plasma and post-operative plasma metabolites were altered (post-operative metabolite level/preoperative metabolite level). The results showed that for ICF patients, 13 metabolites were significantly downregulated (FC < 2, *p* < 0.05) and 94 metabolites were significantly upregulated (FC > 2, *p* < 0.05) after LVAD support (Figure 4A and Table S4). 

**Figure 5:**
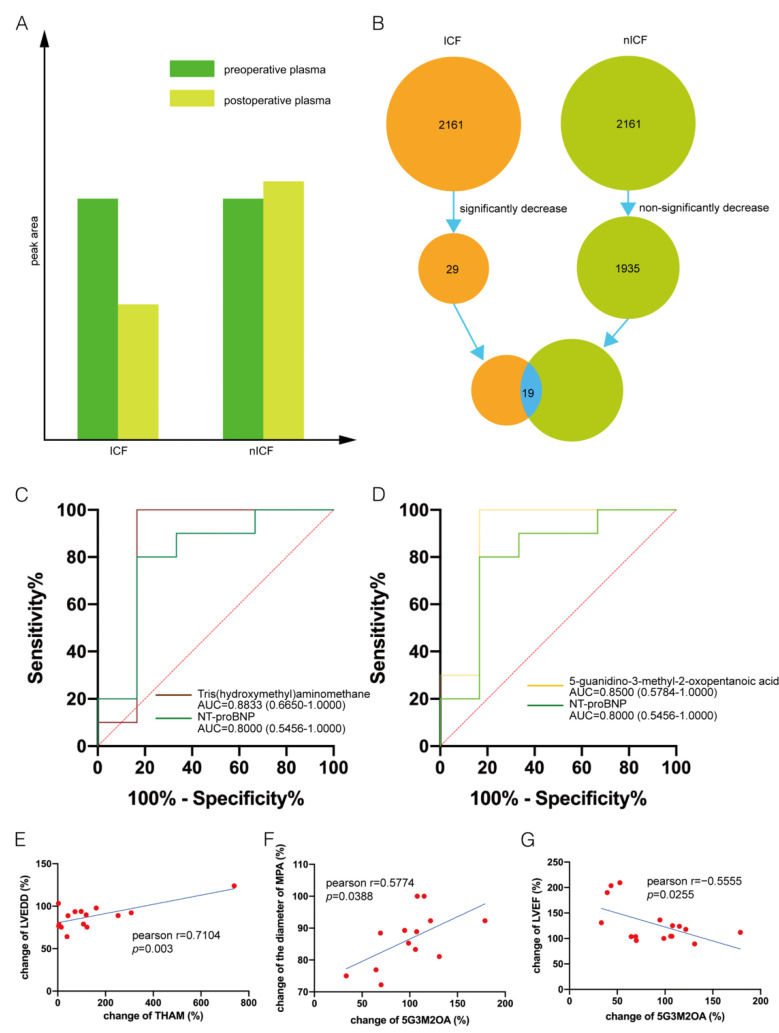
Biomarker identification: (**A**,**B**). The screening pipeline of biomarkers identification. (**C**). Receiver operating characteristic (ROC) curve of Tris(hydroxymethyl)aminomethane (THAM). (**D**). ROC curve of 5-guanidino-3-methyl-2-oxopentanoic acid (5G3M2OA). (**E**). Correlations between the change of THAM (post-operative plasma vs. preoperative plasma) and the change of left ventricular end-diastolic diameter. (**F**). Correlations between the change of 5G3M2OA and the change of diameter of the main pulmonary artery. (**G**). Correlations between the change of 5G3M2OA and the change of left ventricular ejection fraction.

For nICF patients, 135 metabolites were significantly downregulated (FC < 2, *p* < 0.05) and 85 metabolites were significantly upregulated (FC > 2, *p* < 0.05) after LVAD support (Figure 4B and Table S4). We hypothesized that candidate biomarkers would significantly decrease in ICF patients after LVAD support but not in the nICF patients (Figure 5A). 

We screened the predictive biomarkers using the following 4 steps: (1) Screening for significantly decreased metabolites in the ICF group post-operatively: a total of 29 metabolites met this criterion. (2) Screening for metabolites that did not significantly decrease in nICF patients post-operatively: a total of 1935 metabolites met this criterion. (3) Screening for metabolites that overlapped in steps 2 and 3: a total of 19 metabolites met this criterion. (4) Selecting metabolites with AUC > 0.8 (Figure 5B). Using strict selection criteria, we identified tris(hydroxymethyl)aminomethane (THAM, AUC, 0.8833; *p* = 0.0227) and 5-guanidino-3-methyl-2-oxopentanoic acid (5G3M2OA, AUC, 0.8500; *p* = 0.0126) as potential biomarkers. In addition, the efficacy of THAM and 5G3M2OA was better than NT-proBNP (AUC, 0.8000; *p* = 0.0509) (Figure 5C,D). Furthermore, the change ratio (CR, post-operation/pre-operation) of THAM positively correlated with the CR of LVEDD (Figure 5E), and CR of 5G3M2OA positively correlated with the diameter of the main pulmonary artery (MPA) (Figure 5F) and negatively correlated with LVEF (Figure 5G), respectively.

## 4. Discussion

Although LVAD support achieved LV unloading with a beneficial effect on symptoms, a variable response was observed in our patients [16,17]. To date, no studies have developed a predictive biomarker for early identification of patients’ response to LVAD support in a high throughput manner. In this study, we used untargeted metabolomic approaches to identify the different metabolites between ICF and nICF patients at baseline and changes in metabolites after LVAD support. Furthermore, we found the metabolic characteristic of ICF and nICF patients and identified two metabolites that could predict the outcome of HF patients after LVAD support (Figure 1). 

A strength of our study was the prospective enrollment of patients who accepted LVAD support, which reduced potential bias attributable to medications. Some studies have reported a decrease in fatty acid metabolism during the progression of HF [18], but there is a lack of consensus regarding the changes in carbohydrate metabolism during HF. Since most of the carbohydrate oxidation levels need to be checked invasively [7], most of the current information on the alteration of carbohydrate metabolism during HF is obtained from the animal level [19]. There has been a large variation in the results obtained from animal models. In a transverse aortic constriction (TAC) mouse model, glucose uptake was decreased [20]. However, a post-infarction HF rat model showed enhanced glucose metabolism [21]. Another study using TAC to induce HF in rats found that glucose oxidation levels initially increased, then returned to normal levels during compensatory HF, and decreased when end-stage HF occurred [22]. The above results suggest that animal models are not representative of metabolic changes during human HF. High throughput metabolomics enables an approach for identifying plasma metabolites during the HF process. Based on our metabolomic findings, no fatty acid-related pathways were observed in either group, which was consistent with the findings of the previous study [18]. In the meanwhile, enrichment analysis revealed a significant difference between ICF and nICF patients. Specifically, metabolites upregulated in ICF patients were enriched in carbohydrate metabolism, amino acid metabolism and ATP metabolism (Figure 3M). The reason for this phenomenon might be enhanced carbohydrate and amino acid metabolism provides energy to the heart, which might partially compensate for HF and thus promote the remodeling of cardiac function. However, metabolites upregulated in nICF patients were mainly enriched in hormone metabolism (Figure 3N), thus failing to provide sufficient energy for cardiac contraction.

LVAD support changes patients’ plasma metabolites [19,23]. To date, potential biomarkers of patients’ response to LVAD support have rarely been identified in both human and animal studies. Our study identified two metabolites, THAM and 5G3M2OA, that could predict the patients’ response to LVAD support. THAM is a small molecule metabolite that is weakly alkaline and relieves acidosis [24]. In clinical practice, plasma pH is an important indicator for assessing the prognosis of patients. Acidosis affects the release of Ca^2+^ from the sarcoplasmic reticulum and interferes with the efficiency of the Na-K pump, which finally results in a disturbance in cellular electrolyte balance [25]. We hypothesized that the long-term compensatory elevation of THAM after LVAD support might reflect a state of suppressed cardiac function. 5G3M2OA is a derivative of arginine metabolism [26]. Aspartate aminotransferase specifically catalyzes transformation from the aspartate to the resulting 5G3M2OA, generating β-methylarginine [26]. Arginine is important for maintaining blood pressure (BP) and hemodynamic homeostasis. Experiments in rats demonstrated that BP decreased after intravenous arginine infusion. When the infusion was discontinued, BP gradually recovered [27]. It was reported that changes in amino acid metabolism in patients with LVAD support, and our study further confirmed that the intermediate molecule of the arginine metabolic process, 5G3M2OA, was maintained at high levels and might affect the recovery of cardiac function in patients after LVAD support.

The ideal biomarker for predicting response to LVAD support would have high sensitivity and specificity in a stable and rapid manner. To date, however, few biomarkers are applicable. Although plasma NT-proBNP is widely used in evaluating cardiac function clinically, it only reflects the current state of cardiac function. Our metabolites-based predictive biomarkers may be useful for early prediction of response to LVAD support. Identifying changes in metabolites in the early post-operative period (within 1 week) can predict the recovery of cardiac function 6 months after surgery, thus allowing early identification of patients who do not respond to LVAD support. For patients who have difficulty recovering cardiac function, aggressive metabolic intervention and an early search for a donor for HTx are required.

## 5. Conclusions

In summary, the MS-based metabolomic technique is an effective approach to identifying differential features between ICF and nICF patients after LVAD support. The nontargeted metabolomic method is an effective strategy to screen potential predictive biomarkers of patients’ response to LVAD support. Early identification of ICF patients and nICF patients helps surgeons to administer supportive treatments, such as adding nICF patients to the HTx list, in a timely manner. We hope to offer a framework that will fulfill a more precision medicine in HF treatment.

## Figures and Tables

**Table 1:** Clinical characteristics of patients.

Variable	ICF (*n* = 6)	nICF (*n* = 10)	*p-*Value
Age at implantation	39.2 (30.50–46.25)	40.5 (30.75–47.75)	0.8125
Male sex	6 (100%)	9 (90%)	0.4577
HF etiology			0.2769
ICM	2 (33%)	1 (10%)	
DCM	4 (67%)	6 (60%)	
VHD	0	2 (20%)	
ACM	0	1 (10%)	
BMI (kg/m^2^)	25.5 ± 4.27	23.3 ± 5.01	0.4167
NYHA functional class			0.4250
I	0	0	
II	1 (16.7%)	0	
III	0	1 (10%)	
IV	5 (83.3%)	9 (90%)	
Diabetes	0	1(10%)	0.4577
Creatinine (μmol/L)	87.4 ± 28.47	99.4 ± 19.48	0.3324
Hemoglobin (g/L)	115.3 ± 28.65	135.9 ± 15.47	0.0805

ICF: improved cardiac function; nICF: non-improved cardiac function; ICM: ischemic cardiomyopathy; DCM: dilated cardiomyopathy; VHD: valvular heart disease; ACM: alcoholic cardiomyopathy.

## Data Availability

Not applicable.

## Supplementary Materials

The following supporting information can be downloaded at: https://www.mdpi.com/article/10.3390/metabo12111068/s1, Table S1: Preoperative metabolites; Table S2: 714 endogenous metabolites; Table S3: Significantly different metabolites nICF pre LVAD vs. ICF pre LVAD; Table S4: Significantly different metabolites pre LVAD vs. post LVAD
